# A meta-analysis of the dose–response relationship between aerobic exercise and executive function in children

**DOI:** 10.3389/fpubh.2025.1608937

**Published:** 2025-12-09

**Authors:** Yanhua Wang, Qi Wang, Min Yang, Wenwen Li, Lu Wang, Jiaqi Fan

**Affiliations:** 1 Department of PE, Civil Aviation University of China, Tianjin, China; 2 School of Physical Education, Yanshan University, Qinhuangdao, China; 3 Institute of Physical Education, Kunming University of Science and Technology, Kunming, China

**Keywords:** cognitive flexibility, inhibitory control, physical activity, subgroup analysis, working memory

## Abstract

**Objective:**

While existing meta-analyses of executive function focus on older adults and clinical pediatric populations, a substantial gap remains regarding typically developing children during this critical period. Our meta-analysis elucidates the dose–response relationship between aerobic exercise and executive function in this group, providing an evidence-based framework for exercise prescription and translational research.

**Methods:**

A comprehensive literature search was conducted to retrieve 4,135 relevant articles examining the impact of aerobic exercise on children’s executive function from multiple databases, including PubMed, Embase, Web of Science, Cochrane Library, China Biology Medicine Database (CBM), CNKI, Wanfang, and VIP. The search period spanned from the inception of each database to December 2024. The quality of the included literature was assessed using the ROB 2 tool, and data analysis was conducted using RevMan 5.4 and Stata 16 software. Furthermore, the GRADE evidence quality evaluation tool was employed to assess the outcome indicators of the included studies.

**Results:**

A total of 19 articles were ultimately included, consisting of 17 English-language articles and 2 Chinese-language articles. All studies were randomized controlled trials (RCTs) involving a total of 18,650 children. The results indicated that the standardized mean differences (SMD) for the effects of aerobic exercise interventions on children’s inhibitory control, working memory, and cognitive flexibility were as follows: SMD = 0.06, 95% CI (−0.09, −0.03) for inhibitory control; SMD = −0.01, 95% CI (−0.04, −0.02) for working memory; and SMD = -0.02, 95% CI (−0.01, −0.05) for cognitive flexibility. According to the GRADE evidence quality assessment, the three outcome indicators (inhibitory control, working memory, and cognitive flexibility) were rated as moderate quality.

**Conclusion:**

Single acute aerobic exercise sessions lasting less than 30 min demonstrate the most significant improvement in executive function among children aged 6 to 8 years.

**Systematic review registration:**

https://www.crd.york.ac.uk/PROSPERO/view/CRD42025631031, identifier (CRD42025631031).

## 1 Introduction

Executive function (EF) refers to the higher-order cognitive processes that coordinate and control various basic cognitive functions during the completion of complex cognitive tasks (1). It encompasses three sub-functions, namely inhibitory control, working memory, and cognitive flexibility (2). During the development process from early childhood to adolescence in children, executive function exhibits the most vigorous development in the primary school stage (6–12 years old) (3), and has a close relationship with aspects such as individual physical and mental health (4), academic performance (5, 6), socialization development (7), and emotional control, among others (8).

There exist numerous approaches for promoting the development of executive function, such as cognitive training, pharmacological intervention, exercise intervention, multiple combined interventions, etc. (9). Among them, exercise intervention is a mode that is simple to operate, economical and free of side effects. Aerobic exercise refers to physical activities that predominantly utilize aerobic metabolism for energy production, which is a form of endurance exercise with the objective of improving the body’s capacity to inhale, transport, and utilize oxygen (10). Literature indicates that aerobic exercise training is more sensitive to executive function compared to other aspects of cognition (11). However, some studies have found no significant impact of aerobic exercise on children’s executive function (12–14). A review of meta-analyses on executive function reveals that most studies have concentrated on the older adults (11, 15, 16) and children with impaired executive function (17–19). In contrast, comparatively limited attention has been devoted to children in the critical developmental period for executive function. This study employs a meta-analytic approach to elucidate the underlying mechanisms and key moderating variables through which aerobic exercise influences children’s executive function, and to further examine the dose–response relationship between the two. The findings aim to inform the development of targeted aerobic exercise prescriptions for children, thereby establishing a robust evidence-based foundation for future research and the implementation of exercise interventions aimed at enhancing executive function in this population.

## 2 Materials and methods

### 2.1 Protocol

This meta-analysis was conducted in accordance with the Preferred Reporting Items for Systematic Reviews and Meta-Analyses (PRISMA) guidelines. The review has been registered with the International Prospective Register of Systematic Reviews (PROSPERO) with the registration number CRD42025631031.

### 2.2 Search strategy

Relevant literature were searched through computer in the electronic databases of PubMed, Embase, Cochrane Library, Web of Science, China Biology Medicine Database (CBM), CNKI, VIP, and Wanfang databases from their inception to December 2024. The search terms included: “Executive Function,” “Executive Control,” “Central Executive,” “Cognition,” “Cognitive Flexibility,” “Inhibition,” “Working Memory,” “Children,” “Adolescent,” “Teenager,” “Middle Childhood,” “Adolescence,” “Youth,” “Exercises,” “Physical Activity,” “Acute Exercise,” “Exercise Isometric,” “Randomized Controlled Trial,” “RCT,” etc. Taking PubMed as an example, the specific retrieval strategy was described in BOX 1.

BOX 1 **PubMeb search strategy**

**Table:** 

#1	“Executive Function” (Mesh)
#2	Executive Functions OR Function Executive OR Functions Executive OR Executive Control OR Executive Controls OR Central Executive OR Cognition OR Cognitive Flexibility OR Cognitive Function OR Cognitive Performance OR Inhibition OR Response Time OR Working Memory OR Task Switching
#3	#1 OR #2
#4	Children OR Child OR Kid OR Adolescent OR Teenager OR School Children OR Middle Childhood OR Preadolescence OR Adolescence OR Youth
#5	#3 AND #4
#6	“Aerobic exercise” (Mesh)
#7	Exercises OR Physical Activity OR Activities, Physical OR Activity, Physical OR Physical Activities OR Exercise, Physical OR Exercises, Physical OR Physical Exercise OR Physical Exercises OR Acute Exercise OR Acute Exercises OR Exercise, Acute OR Exercises, Acute OR Exercise, Isometric OR Exercises, Isometric OR Isometric Exercises OR Isometric Exercise OR Exercise, Aerobic OR Aerobic Exercise OR Aerobic Exercises OR Exercises, Aerobic OR Exercise Training OR Exercise Trainings OR Training, Exercise OR Trainings, Exercise
#8	#6 OR #7
#9	randomized controlled trial[Publication Type] OR randomized OR placebo
#10	#5 AND #8 AND #9

### 2.3 Inclusion and exclusion criteria

#### 2.3.1 Inclusion criteria

① Study subjects: Children aged 6–12 years, regardless of gender; ② Intervention measures: Various types of aerobic exercise; ③ Control measures: No exercise intervention or non-aerobic exercise; ④ Outcome indicators: At least one indicator of executive function, inhibitory control, working memory, or cognitive flexibility, with data presented as mean (M) and standard deviation (SD); ⑤ Study type: Randomized controlled trials (RCTs).

#### 2.3.2 Exclusion criteria

① Non-intervention studies; ② Non-experimental or animal studies; ③ Studies with insufficiently described interventions; ④ Studies targeting non-healthy children and adolescents; ⑤ Studies from which data on executive function, inhibitory control, working memory, or cognitive flexibility cannot be extracted; ⑥ Review articles, duplicate publications; ⑦ Unpublished studies, abstracts, or theses.

### 2.4 Data extraction

Two researchers independently screened the literature according to the inclusion and exclusion criteria. The initial screening was based on the titles and abstracts. Full-text articles were then downloaded and carefully read. Information extracted for analysis included the author, country, publication year, study subjects, intervention content, intervention protocol (duration, frequency, and period), and outcome indicators. Cross-checking was performed to ensure accuracy. Discrepancies were resolved through discussion between the two researchers, and if consensus could not be reached, a third researcher was consulted to make the final decision.

### 2.5 Quality assessment of literature

The Risk of Bias tool (ROB 2) was employed to evaluate the risk of bias around the following five indicators: randomization process, deviations from intended interventions, missing outcome data, measurement of outcomes, and selective reporting of results. Each indicator was evaluated based on the fulfillment of the criteria, and the risk of bias was judged as “yes,” “probably yes,” “probably no,” “no,” or “no information.” Two researchers independently scored and assessed the studies, and any discrepancies were resolved through discussion with a third researcher to reach a final consensus.

### 2.6 Statistical analysis

Meta-analysis was conducted using Stata 16.0 and RevMan 5.4 software. The outcome data were continuous variables. When the measurement tools and units were identical, the weighted mean difference (WMD) was used; otherwise, the standardized mean difference (SMD) was employed to eliminate the different units and merge the results. The 95% confidence interval (CI) was used as the effect size indicator. Heterogeneity was assessed using the I^2^ test. If I^2^ < 50% and *p* ≥ 0.1, indicating low heterogeneity, a fixed-effect model was used; otherwise, a random-effect model was applied (I^2^ ≥ 50% and *p* < 0.1). Sensitivity analysis and subgroup analysis were conducted to identify potential sources of heterogeneity. Sensitivity analysis was performed by sequentially excluding each study; if the heterogeneity did not substantially decrease, the results were deemed robust. Subgroup analyses were conducted based on participant age (6–8 years, 8–10 years, 10–12 years), intervention frequency (single acute exercise, twice per week, three times per week), intervention duration (30 min, 40–55 min, 60 min or more), and intervention period (single acute exercise, 8–12 weeks, more than 12 weeks). Funnel plots were created for the outcome indicators, and Egger’s test was used to assess publication bias.

### 2.7 GRADE evidence quality assessment

The GRADE system is a rigorous tool for evaluating the quality of clinical RCT evidence, known for its transparency and practicality (20). The GRADE profiler was used to assess the evidence quality, which is categorized into four levels: high, moderate, low, and very low. The assessment considered five aspects: risk of bias, inconsistency, indirectness, imprecision, and publication bias.

High: The true effect is close to the estimated effect, with minimal impact from further studies. Moderate: The true effect is likely to be close to the estimated effect, but there is a possibility of differences, and further studies may have a significant impact. Low: Confidence in the estimated effect is limited, and the true effect may be substantially different from the estimated effect, with further studies likely to influence or even change the results. Very low: The true effect is likely to be different from the estimated effect, with high uncertainty in the results.

## 3 Results

### 3.1 Literature screening results

**Figure 1:**
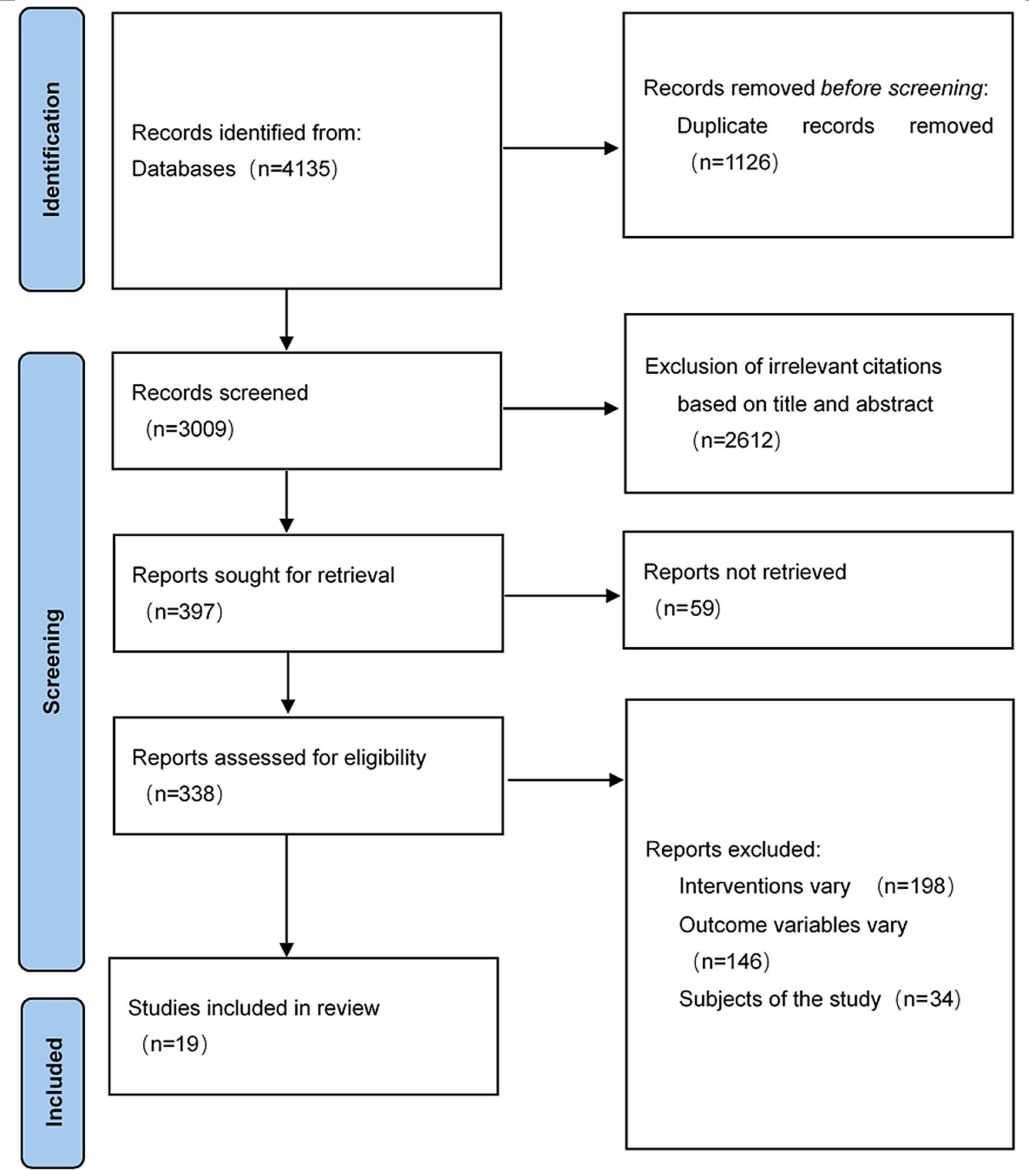
Flowchart of literature screening.

Using the predefined search strategy, a total of 4,135 relevant articles were retrieved from PubMed (608), Embase (217), Cochrane Library (1,851), Web of Science (1,000), China Biology Medicine Database (113), CNKI (202), VIP (21), and Wanfang (107). Among these articles, 1,126 duplicate records were removed by using EndNote X9, and 2,612 articles were excluded after reading the titles and abstracts, leaving 397 articles for full-text assessment. Of these, 198 were excluded due to inconsistent interventions, 146 due to inconsistent outcome variables, and 34 due to mismatched study subjects. Ultimately, 19 articles were included (21–39), with the specific screening process shown in Figure 1.

### 3.2 Basic characteristics of the included studies

A total of 19 studies were included, comprising 17 English articles (21–37) and 2 Chinese articles (38, 39), involving 18,673 children. Inhibitory control was measured in 17 studies (21–25, 27–36, 38, 39), working memory in 12 studies (21–23, 25–27, 30, 31, 36–39), and cognitive flexibility in 8 studies (22, 23, 27, 31, 36–39). All included studies involved children aged 6–12 years. Among the 19 studies, 6 were single acute exercise interventions (21, 22, 27, 29, 35, 39), 5 had intervention periods of 8–12 weeks (23, 31, 33, 34, 37), and 8 had intervention periods longer than 12 weeks (24–26, 28, 30, 32, 36, 38). The basic characteristics of the included studies are shown in Table 1.

**Table 1:** Basic characteristics of the included studies.

First author (year)	Country (language)	Age (years)	Simple size (T/C)	Intervention protocol		Frequency/duration/period	Outcome indicators
				T	C		
Berg (34) (2019)	Netherlands (English)	10.8 ± 0.6	263/249	Dancing	Video	Once per week; 10–15 min/session; 9 weeks	4.
Bruijn (26) (2022)	Netherlands (English)	9.22 ± 0.72	22/17	physical activity	Physical Education class	Four times per week; 30 min/session; 14 weeks	6.
Chen (22) (2014)	China (English)	9.24 ± 0.44 11.07 ± 0.27	44/43	jogging	reading	Single acute exercise; 30 min	1.2.3.
Cheng (23) (2023)	China (English)	7.5 ± 0.5	12/12	Equine Assisted Therapy	Physical Education class	Twice per week; 45–55 min/session; 12 weeks	1.2.3.
Cho (24) (2017)	South Korea (English)	11.20 ± 0.77	15/15	Taekwondo	Physical Education class	Five times per week; 60 min/session; 16 weeks	4.
Condello (25) (2021)	Italy (English)	10.73 ± 0.32	91/90	Aerobic exercise	Physical Education class	Once per week; 60 min/ session; 24 weeks	5.
Cooper (27) (2018)	Britain (English)	12.3 ± 0.7	39/39	basketball	None	Single acute exercise; 60 min	4.7.8.
Drollette (28) (2018)	United States (English)	8.72 ± 0.05	139/169	physical activity	None	Five times per week; at least 70 min/session; 9 months	1.
Irene (35) (2020)	Netherlands (English)	8.81 ± 0.6	23/47	Aerobic exercise	None	Single acute exercise; 35 min	9.
Lind (29) (2019)	Denmark (English)	11.8	27/27	football	None	Single acute exercise; 20 min	1.
Meijer (30) (2021)	Netherlands (English)	9.30 ± 0.65	206/415	aerobic exercise	Physical Education class	Four times per week; 30 min/session; 14 weeks	9.10.
Purohit (31) (2017)	India (English)	12.58 ± 1.35	40/32	Yoga	None	Four times per week;90 min/session; 3 months	4.8.11.
Roh (32) (2018)	South Korea (English)	11.53 ± 0.64	15/15	Taekwondo	None	Once per week; 60 min/session; 16 weeks	4.
St Laurent (33) (2019)	United States (English)	8.8 ± 0.1	27/29	Comprehensive exercises	None	Five times per week; 15 min/session; 3 months	1.12.
Wassenaar (36) (2021)	Britain (English)	12.5 ± 0.296	7,860/8,157	HIIT	None	Twice per week; 60 min/session; 10 months	1.2.13.
Wen (21) (2021)	China (English)	10.9 ± 0.3	67/37	football	Movie	Single acute exercise; 40 min	2.14.
Yan (39) (2014)	China (Chinese)	9.81 ± 0.26	80/40	calisthenics, steeplechase	None	Single acute exercise; 30 min	1.2.3.
Yin (38) (2017)	China (Chinese)	9–10	36/21	basketball	None	Three times per week; 30 min/session; 16 weeks	1.2.3.
Zask (37) (2022)	Australia (English)	9.8 ± 0.10	101/89	MVPA	None	Five times per week; 30 min/session; 6 weeks	8.

Outcome indicators: 1. Flanker; 2. N-Back; 3. More-odd shifting; 4. Stroop test; 5. Random Number Generation (RNG); 6. Spatial Span task; 7. Sternberg paradigm; 8. Trail making test; 9. Stop Signal Task; 10. Digit Span Task; 11. Wechsler intelligence scale for children; 12. List Sorting Working Memory Test; 13. colour-shape switching task; 14. Go/NoGo.

### 3.3 Quality assessment of included studies

**Figure 2:**
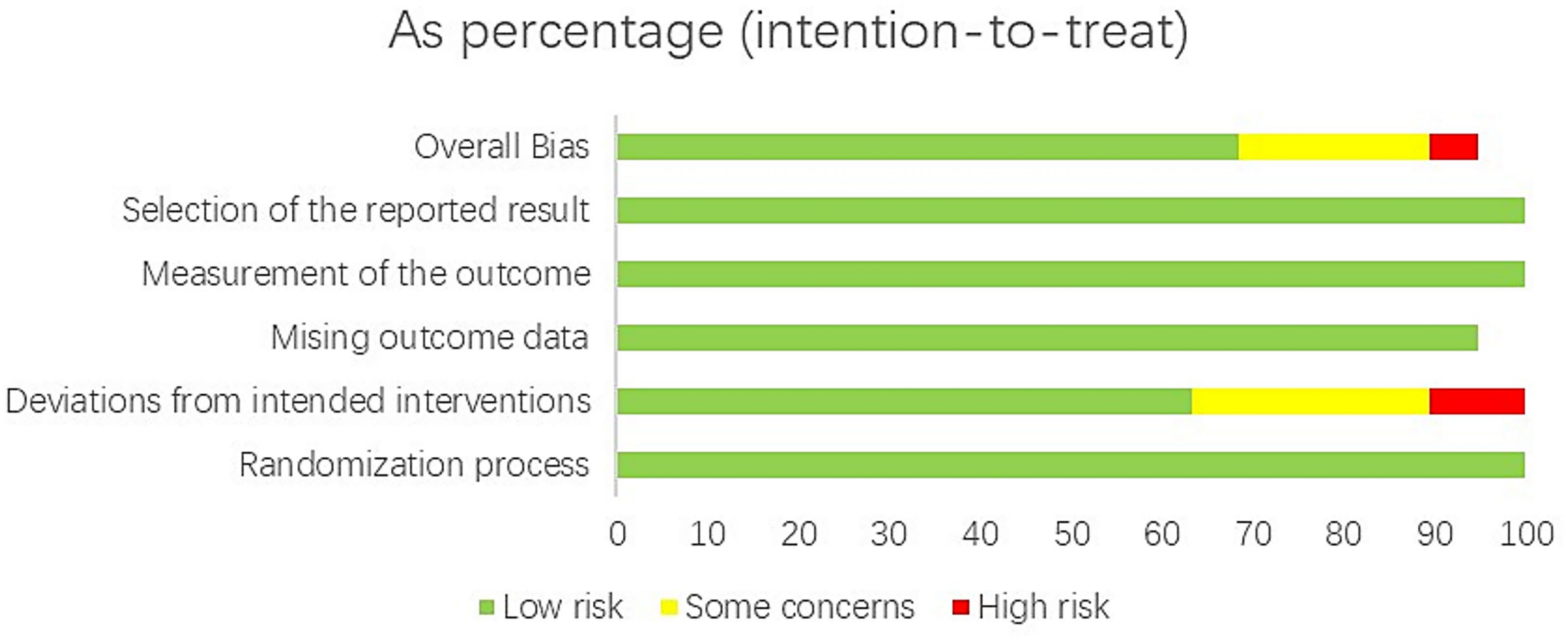
Bias risk assessment of included studies.

**Figure 3:**
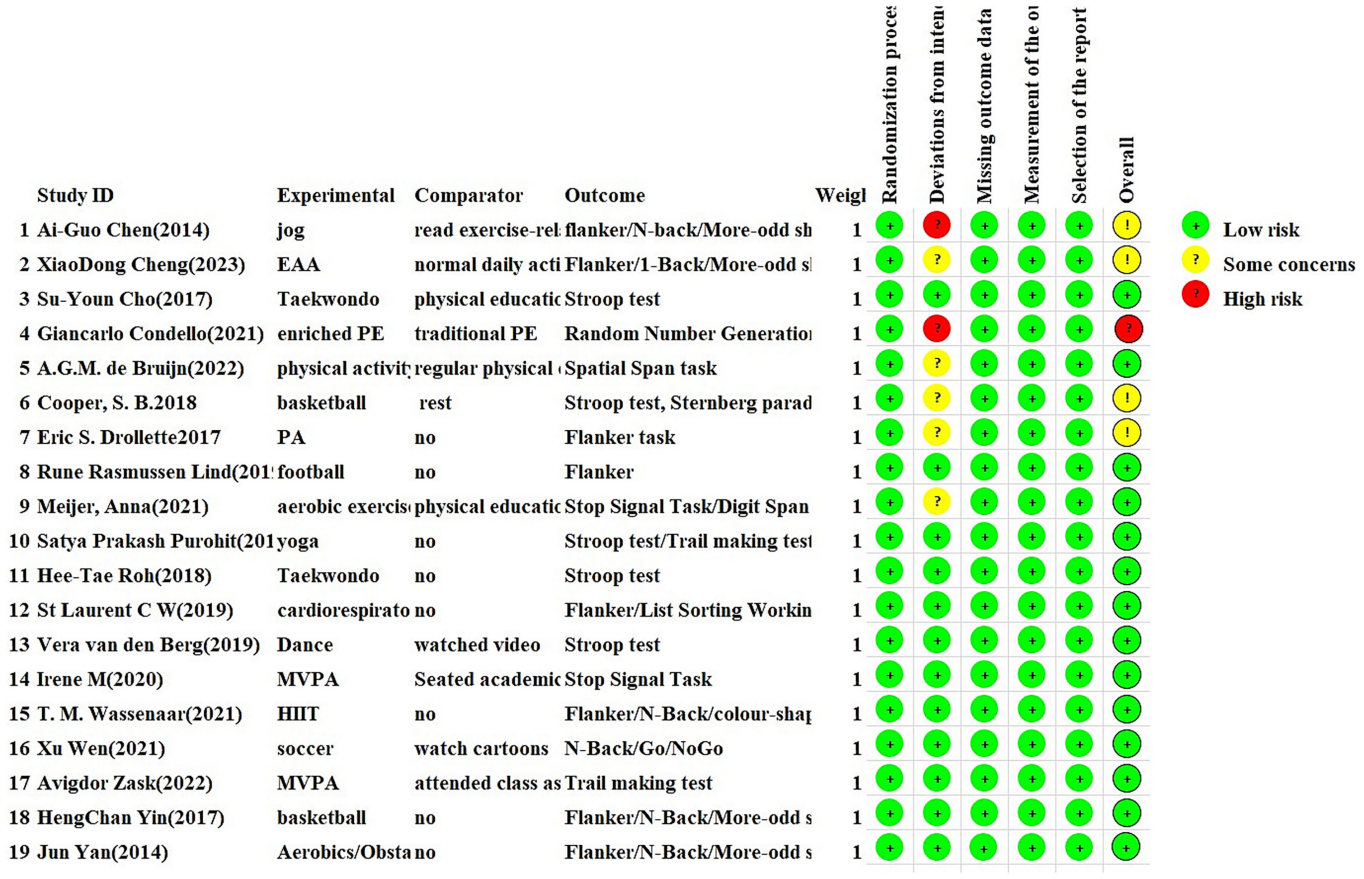
Summary of bias risk of included studies.

The methodological quality assessment aims to evaluate whether the included studies in the meta-analysis adhere to scientific standards in terms of study design and process. High-quality methodological evaluation can effectively reduce the bias between actual study results and published results, ensuring the authenticity and reliability of the meta-analysis results (40). The 19 included RCTs were assessed using the ROB 2.0 tool across five domains. Among these, four RCTs were identified as having a potential risk of bias (22, 23, 27, 28), 14 RCTs were classified as having a low risk of bias (21, 24, 26, 29–39), and one RCT was classified as having a high risk of bias (25). In the assessment of bias risk due to deviations from intended interventions, five RCTs were identified as having a potential risk of bias (23, 26–28, 30), 12 were classified as having a low risk of bias (21, 24, 29, 31–39), and two were assessed as having a high risk of bias due to serious deviations (22, 25). No risk of bias was identified in the randomization process, missing outcome data, outcome measurement, and selective reporting of results (see Figures 2, 3).

### 3.4 Meta-analysis results

#### 3.4.1 Impact of aerobic exercise on inhibitory control

**Figure 4:**
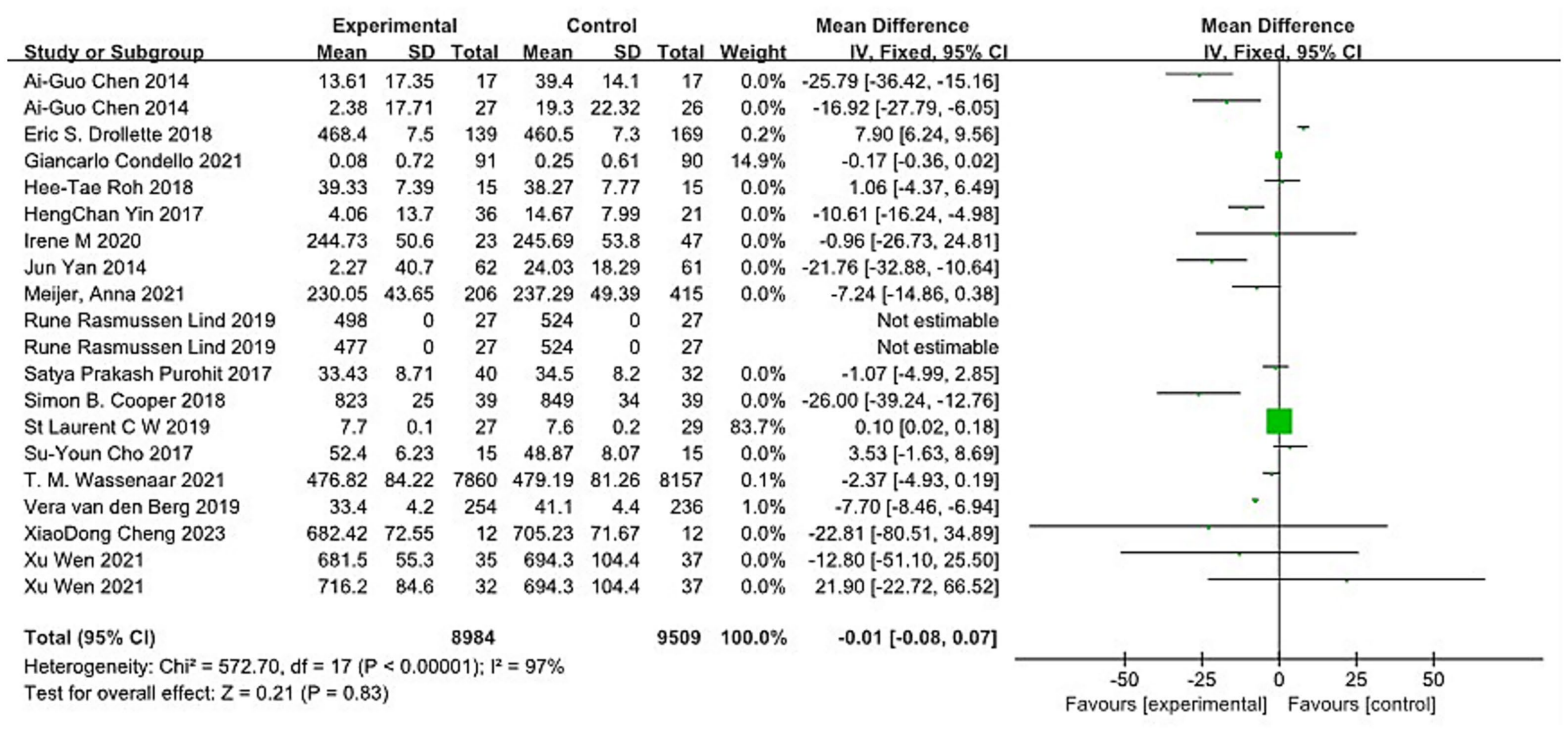
Forest plot of the impact of aerobic exercise on children’s inhibitory control.

A total of 17 articles reported 20 experiments on the impact of aerobic exercise on children’s inhibitory control, involving a total sample of 18,493 participants (8,984 in the aerobic exercise group and 9,509 in the control group). Heterogeneity tests were conducted on the 20 included studies, and significant heterogeneity was found among the studies (I^2^ = 97%, *p* < 0.01). A random-effects model was used to synthesize the effect size, resulting in SMD = −0.01, 95% CI (−0.08, −0.07), p < 0.01 (Figure 4). The difference was statistically significant, indicating that aerobic exercise intervention significantly promotes inhibitory control in children. Due to the significant heterogeneity among the included studies. Sensitivity analysis was conducted by excluding each study one by one. The results showed that the overall effect size was not significantly affected by the removal of any single study, indicating robustness of the meta-analysis results.

#### 3.4.2 Impact of aerobic exercise on working memory

**Figure 5:**
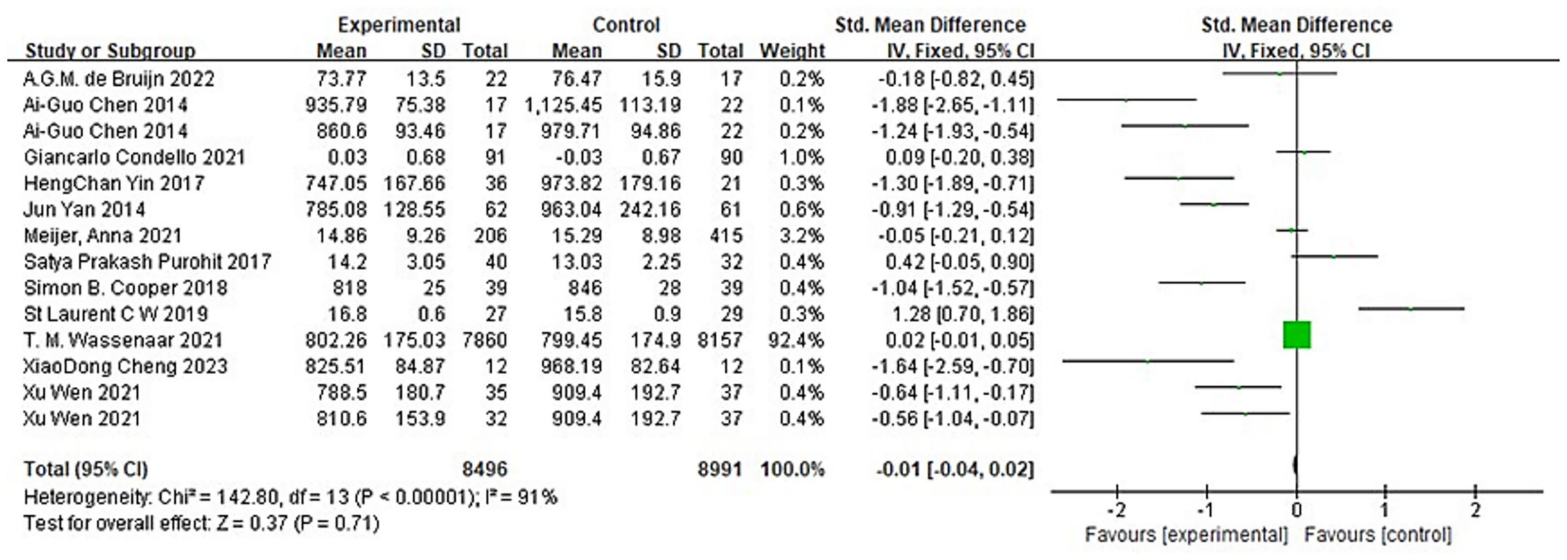
Forest plot of the impact of aerobic exercise on children’s working memory.

A total of 12 articles reported 14 experiments on the impact of aerobic exercise on children’s working memory, involving a total sample of 17,492 participants (8,501 in the aerobic exercise group and 8,991 in the control group). Heterogeneity tests were conducted on the 14 included studies, and significant heterogeneity was found among the studies (I^2^ = 91%, P<0.00001). A random-effects model was used to synthesize the effect size, resulting in SMD = -0.01, 95%CI (−0.04, −0.02), *p* < 0.01 (Figure 5). The difference was statistically significant, indicating that aerobic exercise intervention significantly promotes working memory in children. Due to the significant heterogeneity among the included studies. Sensitivity analysis was conducted by excluding each study one by one. The results showed that the overall effect size was not significantly affected by the removal of any single study, indicating robustness of the meta-analysis results.

#### 3.4.3 Impact of aerobic exercise on cognitive flexibility

**Figure 6:**
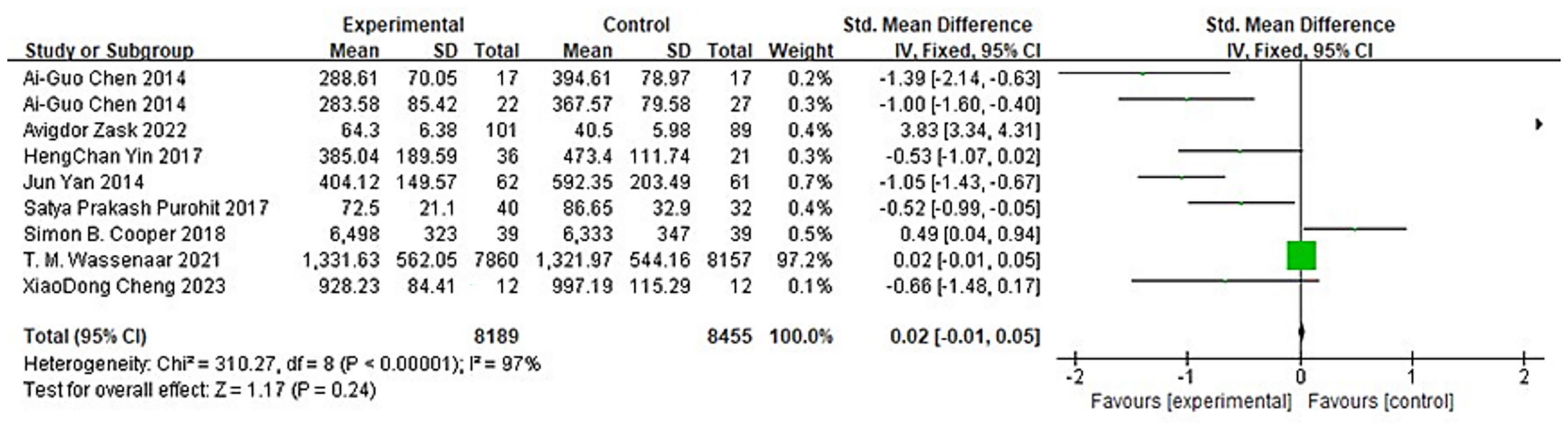
Forest plot of the impact of aerobic exercise on children’s cognitive flexibility.

A total of 8 articles reported 9 experiments on the impact of aerobic exercise on children’s cognitive flexibility, involving a total sample of 16,644 participants (8,189 in the aerobic exercise group and 8,455 in the control group). Heterogeneity tests were conducted on the 9 included studies, and significant heterogeneity was found among the studies (I^2^ = 97%, P<0.01). A random-effects model was used to synthesize the effect size, resulting in SMD = -0.02, 95%CI (−0.01, −0.05); P<0.01 (Figure 6). The difference was statistically significant, indicating that aerobic exercise intervention significantly promotes cognitive flexibility in children. Due to the heterogeneity among the included studies. Sensitivity analysis was conducted by excluding each study one by one. The results showed that the overall effect size was not significantly affected by the removal of any single study, indicating robustness of the meta-analysis results.

In summary, the meta-analysis of the three subfunctions of executive function revealed that aerobic exercise intervention positively promotes the development of children’s executive function. However, relatively high heterogeneity was observed within each group. Therefore, further subgroup analyses were conducted to explore the impact of exercise intervention period, duration, frequency, and age on the intervention effects.

### 3.5 Subgroup analysis

To further investigate the impact of aerobic exercise on children’s executive function, subgroup analyses were conducted based on four moderators: exercise frequency, duration, period, and age (Tables 2–4). Exercise frequency was divided into single acute exercise, three times per week or less, and more than three times per week. Exercise duration was divided into less than 30 min, 40–55 min, and 60 min or more. Exercise period was divided into single acute exercise, 8–12 weeks, and more than 12 weeks. Age was divided into 6–8 years, 8–10 years, and 10–12 years. Heterogeneity was considered relatively significant if I^2^ > 50%, and *p* < 0.05 indicated a significant difference.

- The subgroup analysis of exercise frequency revealed that the effects of aerobic exercise intervention are influenced by the frequency of exercise. Single acute exercise sessions demonstrated the most significant improvement in children’s inhibitory control (SMD = −0.47, *p* < 0.05) and working memory (SMD = −0.92, p < 0.05). In contrast, exercising more than three times per week showed the greatest improvement in cognitive flexibility (SMD = 1.61, *p* < 0.05).
- The subgroup analysis of exercise duration revealed that the effects of aerobic exercise intervention are influenced by the duration of each exercise session. Exercise sessions lasting less than 30 min demonstrated the most significant improvement in children’s inhibitory control (SMD = −1.47, *p* < 0.05) and working memory (SMD = 1.28, *p* < 0.05). In contrast, exercise sessions lasting 30–60 min showed the greatest improvement in cognitive flexibility (SMD = −0.06, *p* < 0.05).
- The subgroup analysis of intervention period revealed that single acute exercise sessions demonstrated the most significant improvement in inhibitory control (SMD = −1.22, p < 0.05) and working memory (SMD = −0.92, p < 0.05). In contrast, an 8–12 week intervention period showed the greatest improvement in cognitive flexibility (SMD = 1.29, p < 0.05).
- The subgroup analysis of age revealed that aerobic exercise showed the best improvement in children aged 6–8 years for inhibitory control (SMD = 0.78, p < 0.05), working memory (SMD = −1.64, p < 0.05), and cognitive flexibility (SMD = −0.66, p < 0.05).

**Table 2:** Subgroup analysis results for inhibitory control.

Moderator variable	Subgroup category	Quantity of literature	SMD (95%CI)	I^2^		*p*
Frequency	Single acute exercise	6	−0.47 (−0.65, −0.29)	78%	96%	0.00
	Three times per week or less	6	−0.07 (−0.10, −0.04)	98%		0.00
	More than three times per week	5	0.25 (0.12, 0.37)	94%		0.00
Duration	Less than 30 min	3	−1.47 (−1.67, −1.28)	99%	96%	0.00
	30–60 min	7	−0.29 (−0.41, −0.16)	75%		0.00
	60 min or more	7	−0.02 (−0.05, 0.01)	94%		0.00
Period	Single acute exercise	6	−0.47 (−0.65, −0.29)	78%	96%	0.00
	8–12 weeks	4	−1.22 (−1.40, −1.05)	97%		0.00
	More than 12 weeks	7	−0.02 (−0.05, 0.01)	94%		0.00
Age	6–8 years	3	0.78 (0.58, 0.99)	91%	96%	0.00
	8–10 years	7	−0.67 (−0.78, −0.56)	97%		0.10
	10–12 years	7	−0.03 (−0.06, −0.00)	70%		0.00

**Table 3:** Subgroup analysis results for working memory.

Moderator variable	Subgroup category	Quantity of literature	SMD (95%CI)	I^2^		*p*
Frequency	Single acute exercise	4	−0.92 (−1.12, −0.72)	52%	91%	0.00
	Three times per week or less	4	0.01 (−0.02, 0.04)	90%		0.00
	More than three times per week	4	0.08 (−0.07, 0.23)	86%		0.00
Duration	Less than 30 min	1	1.28 (0.70, 1.86)	-	91%	0.00
	30–60 min	7	−0.41 (−0.54, −0.28)	87%		0.00
	60 min or more	4	0.01 (−0.02, 0.04)	87%		0.00
Period	Single acute exercise	4	−0.92 (−1.12, −0.72)	52%	91%	0.00
	8–12 weeks	3	0.45 (0.11, 0.79)	92%		0.00
	More than 12 weeks	5	0.01 (−0.02, 0.04)	80%		0.00
Age	6–8 years	1	−1.64 (−2.59, −0.70)	-	91%	0.00
	8–10 years	6	−0.22 (−0.35, −0.08)	93%		0.00
	10–12 years	6	0.01 (−0.02, 0.04)	88%		0.00

**Table 4:** Subgroup analysis results for cognitive flexibility.

Moderator variable	Subgroup category	Quantity of literature	SMD(95%CI)	I^2^		*p*
Frequency	Single acute exercise	3	−0.62 (−0.86, −0.37)	91%	97%	0.00
	Three times per week or less	3	0.01 (−0.02, 0.05)	68%		0.00
	More than three times per week	2	1.61 (1.27, 1.95)	99%		0.00
Duration	Less than 30 min	-	-	-	97%	-
	30–60 min	5	0.06 (−0.16, 0.28)	98%		0.00
	60 min or more	3	0.02 (−0.01, 0.05)	78%		0.00
Period	Single acute exercise	3	−0.62 (−0.86, −0.37)	91%	97%	0.00
	8–12 weeks	3	1.29 (0.97, 1.60)	99%		0.00
	More than 12 weeks	2	0.02 (−0.02, 0.05)	74%		0.00
Age	6–8 years	1	−0.66 (−1.48, 0.17)	-	97%	0.12
	8–10 years	4	0.30 (0.05, 0.55)	99%		0.00
	10–12 years	4	0.01 (−0.02, 0.05)	85%		0.00

In summary, the subgroup analysis of aerobic exercise on children’s executive function showed that single acute aerobic exercise sessions lasting less than 30 min had the best effect on improving executive function in children aged 6–8 years.

### 3.6 Publication bias analysis

**Figure 7:**
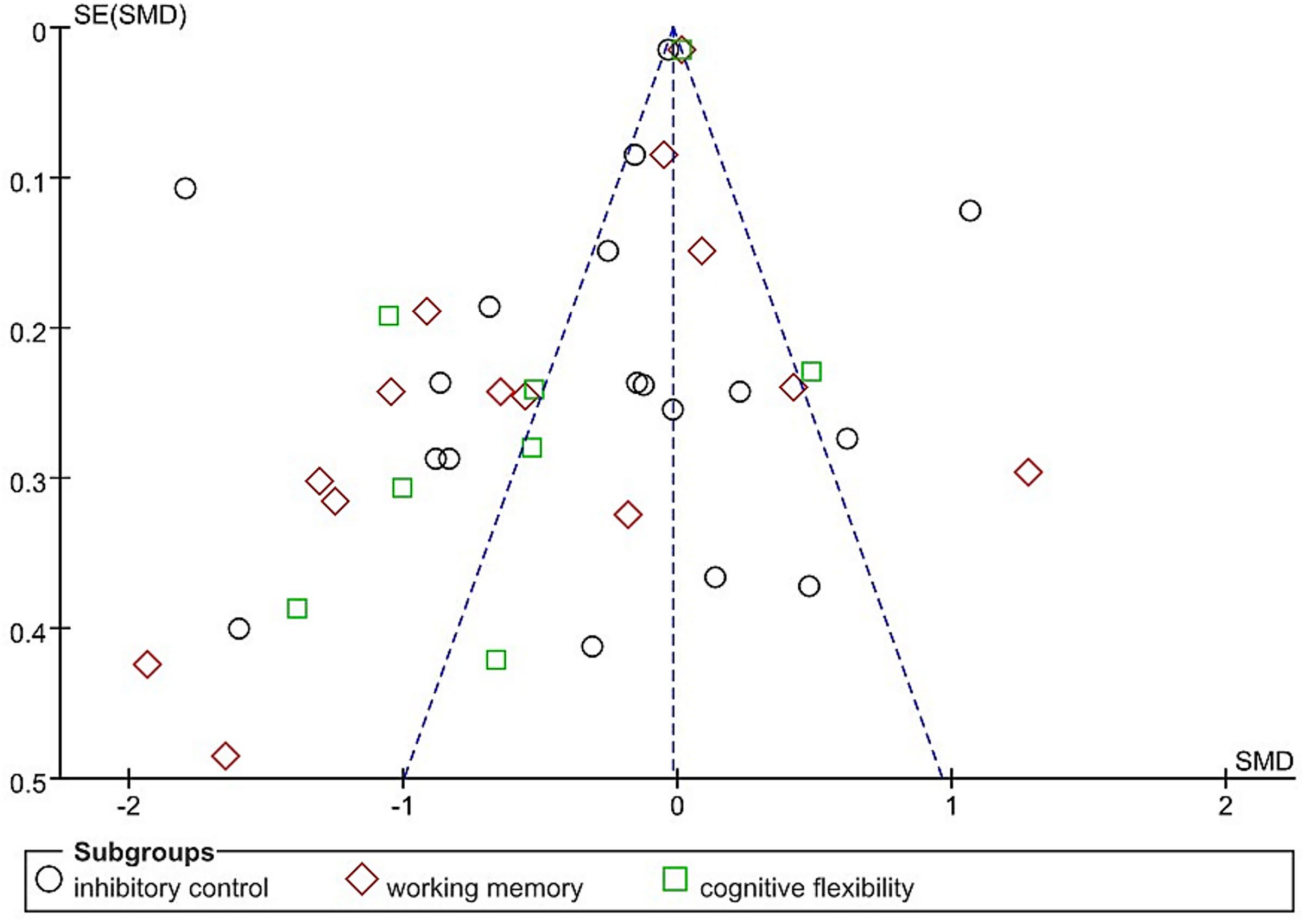
Subgroup analysis results for cognitive function.

Funnel plot was generated using RevMan 5.4 to assess publication bias for the primary outcome indicators of executive function, including inhibitory control, working memory, and cognitive flexibility (see Figure 7). The result revealed that the funnel plot of the outcome indicators was poorly symmetrical. Consequently, Egger’s test was carried out using Stata 16. The findings demonstrated no publication bias for inhibitory control (*p* = 0.4527 > 0.05), working memory (*p* = 0.1614 > 0.05), and cognitive flexibility (*p* = 0.5692 > 0.05).

### 3.7 Evidence quality assessment

The GRADE guidelines were used to assess the quality of evidence for children’s inhibitory control, working memory, and cognitive flexibility. The evidence quality for all three outcomes was rated as “moderate” (see Table 5).

**Table 5:** GRADE quality assessment results.

Outcome indicators (quantity of literature)	Risk of bias	Inconsistency evaluation	Indirectness	Imprecision	Publication bias	Aerobic group	control group	Effect size and confidence interval (95%CI)	Quality result
Inhibitory control (16)	No	Demotion by one level ①	no	no	no	8,961	9,462	−0.06 (−0.09, −0.03)	Moderate
Working memory (12)	No	Demotion by one level ①	no	no	no	8,501	8,991	−0.01 (−0.04, 0.02)	Moderate
Cognitive flexibility (8)	No	Demotion by one level ①	no	no	no	8,189	8,455	−0.02 (−0.04, 0.00)	Moderate

① The heterogeneity test showed a large I^2^ value (75%).

## 4 Discussion

Numerous studies have shown that aerobic exercise can improve children’s executive function (21–39). This study systematically evaluated the impact of aerobic exercise on children’s executive function, particularly on the three core subfunctions: inhibitory control, working memory, and cognitive flexibility. Further subgroup analyses explored the specific effects of different exercise frequencies, durations, periods, and children’s ages on the improvement of each subfunction, aiming to provide a more scientific and personalized exercise prescription for enhancing children’s executive function.

The analysis revealed a significant positive correlation between aerobic exercise and the improvement of children’s executive function, indicating that moderate aerobic exercise can effectively enhance executive function, which is consistent with previous findings (21–39). Unlike previous studies that examined various types of exercise, this study specifically focused on aerobic exercise and explored its dose–response relationship with children’s executive function in greater depth. Specifically, single acute aerobic exercise sessions showed the best improvement in inhibitory control and working memory, while more than three times per week showed the best improvement in cognitive flexibility, consistent with the findings of Graham JD et al. (41). In terms of duration, exercise sessions lasting less than 30 min had the best effect on inhibitory control and working memory, while 30–60 min had the best effect on cognitive flexibility, consistent with the findings of Pontifex MB et al. (42). Regarding the exercise period, single acute exercise had the best effect on inhibitory control and working memory, while an 8–12 week intervention period had the best effect on cognitive flexibility. Additionally, aerobic exercise improved executive function across different age groups, with the most significant effects observed in children aged 6–8 years.

Executive function is a core component of higher-order cognitive functions and is crucial for goal-directed behavior, playing an extremely important role in daily life and mental health (43). For children, the development of executive function is particularly critical, as insufficient or impaired development may lead to learning disabilities, attention deficits, anxiety, depression, schizophrenia and et al. (44). Studies have shown that exercise can improve children’s executive function through several mechanisms, possibly including the followings: (i) During exercise, brain activation necessitates that the increased oxygen supply be proportionate to the elevated neural metabolic demands. However, hyperventilation during intense exercise may lead to decreased cerebral oxygenation and metabolism, interfering with executive function (45–47); (ii) Exercise can increase blood flow velocity, enhance the transport capacity of nutrients in the blood, promote the production of brain-derived neurotrophic factor (BDNF), and activate neuroendocrine pathways, thereby improving executive function (48, 49); (iii) Sustained exercise can lead to vascular survival and differentiation, promote neurogenesis (50). Meanwhile, exercise can induce adaptive structural changes in brain regions closely related to executive function, such as the hippocampus, cerebellum, basal ganglia, and prefrontal cortex, demonstrating the high plasticity of these regions and contributing to the maintenance and enhancement of executive function (51, 52).

The dose–response relationship between physical exercise and executive function in children and adolescents has been extensively validated and is increasingly being incorporated into the development of exercise programs. The dose–response relationship refers to the individual or interactive effects of the various components of physical exercise (type, intensity, period, frequency, and duration) on children’s executive function. This means that different types and intensities of exercise, as well as different frequencies and durations, may have different impacts on children’s executive function. Integrating existing research evidence and the integrative theory of the benefits of physical exercise for children’s executive function, it is evident that when developing exercise programs, both quantitative elements and qualitative factors--such as enriched environments, social interaction, and task complexity--should be considered. Therefore, the formulators of exercise programs should, in accordance with this theory, actively center on the principle of exercise variability and offer exercise situations and tasks that can evoke cognitive challenges for children and adolescents, in order to improve the pertinence and effectiveness of the exercise programs (53). Furthermore, previous systematic reviews and meta-analyses of dose-effect relationships have incorporated more laboratory studies mainly centered on treadmill and body-building vehicle exercises. The variability exercises that offer cognitive challenges are mostly physical activities in a natural environment and mainly focused on the practice of motor skills (54, 55). Moreover, variability exercises are more beneficial for enhancing positive emotions, interest, and attitude towards exercise, as well as increasing confidence and enterprise in sports. Therefore, this study further explored the dose–response relationship of physical exercise benefits for children’s executive function from the perspective of variability exercise. The results showed that aerobic exercise has a promoting effect on children’s executive function, consistent with previous related studies (56–58). Aerobic exercise can improve cardiorespiratory function, enhance physical endurance and adaptability, thereby contributing to the improvement of children’s cognitive and executive functions. However, different types of exercise may have different impacts on different aspects of executive function. Therefore, in the process of formulating exercise regimens, diverse types of sports should be taken into comprehensive consideration, and selections should be made in accordance with the actual circumstances and requirements of children and adolescents. In light of the foregoing discussion, the following principles should be adhered to when formulating exercise programs for children and adolescents: i Take both quantitative and qualitative elements into comprehensive consideration to ensure the all-roundness and efficacy of the exercise program; ii Incorporate variability exercises to augment the cognitive challenges for children and adolescents during exercise; iii Choose appropriate types and intensities of exercise in accordance with the actual circumstances and requirements of children and adolescents; iv Inspire children and adolescents to actively engage in exercise and boost their interest and participation of exercise.

This study has the following limitations: i Relatively high heterogeneity was observed in the combined results of the three subfunctions of executive function; ii The included studies utilized a diverse range of executive function assessment tools, and the inconsistency in these tools likely contributed to the high heterogeneity; iii In several subgroups, the number of included studies was relatively limited. Future research should aim to increase the number of studies in these areas to enhance the robustness of meta-analytic findings and provide more comprehensive evidence for improving children’s executive function.

## 5 Conclusion

Aerobic exercise intervention can effectively enhance children’s executive function. Through subgroup analysis of each outcome indicator, it was discovered that a single acute aerobic exercise intervention of less than 30 min exerted the optimal effect on improving the executive function of children aged 6 to 8. This research demonstrated that aerobic exercise might be an effective strategy for facilitating children’s brain development, possessing certain promotional value. Constrained by the quantity of included studies and the complexity of executive function measurement, the above conclusion still requires verification through more high-quality randomized controlled trials (RCTs).

## Data Availability

The original contributions presented in the study are included in the article/supplementary material, further inquiries can be directed to the corresponding author.
